# Obesity-induced downregulation of miR-192 exacerbates lipopolysaccharide-induced acute lung injury by promoting macrophage activation

**DOI:** 10.1186/s11658-024-00558-w

**Published:** 2024-03-14

**Authors:** Siqi Wu, Wenjing Tang, Ling Liu, Ke Wei, Yin Tang, Jingyue Ma, Hongbin Li, Yichan Ao

**Affiliations:** Department of Anesthesiology, The First Affiliated Hospital of Chongqing Medical University, No 1. YouYi Road, Yuzhong District, Chongqing, 400016 China

**Keywords:** Obesity, Acute lung injury, microRNA, Macrophage activation, m6A, Metabolic stress

## Abstract

**Background:**

Macrophage activation may play a crucial role in the increased susceptibility of obese individuals to acute lung injury (ALI). Dysregulation of miRNA, which is involved in various inflammatory diseases, is often observed in obesity. This study aimed to investigate the role of miR-192 in lipopolysaccharide (LPS)-induced ALI in obese mice and its mechanism of dysregulation in obesity.

**Methods:**

Human lung tissues were obtained from obese patients (BMI ≥ 30.0 kg/m^2^) and control patients (BMI 18.5–24.9 kg/m^2^). An obese mouse model was established by feeding a high-fat diet (HFD), followed by intratracheal instillation of LPS to induce ALI. Pulmonary macrophages of obese mice were depleted through intratracheal instillation of clodronate liposomes. The expression of miR-192 was examined in lung tissues, primary alveolar macrophages (AMs), and the mouse alveolar macrophage cell line (MH-S) using RT-qPCR. m6A quantification and RIP assays helped determine the cause of miR-192 dysregulation. miR-192 agomir and antagomir were used to investigate its function in mice and MH-S cells. Bioinformatics and dual-luciferase reporter gene assays were used to explore the downstream targets of miR-192.

**Results:**

In obese mice, depletion of macrophages significantly alleviated lung tissue inflammation and injury, regardless of LPS challenge. miR-192 expression in lung tissues and alveolar macrophages was diminished during obesity and further decreased with LPS stimulation. Obesity-induced overexpression of FTO decreased the m6A modification of pri-miR-192, inhibiting the generation of miR-192. In vitro, inhibition of miR-192 enhanced LPS-induced polarization of M1 macrophages and activation of the AKT/ NF-κB inflammatory pathway, while overexpression of miR-192 suppressed these reactions. BIG1 was confirmed as a target gene of miR-192, and its overexpression offset the protective effects of miR-192. In vivo, when miR-192 was overexpressed in obese mice, the activation of pulmonary macrophages and the extent of lung injury were significantly improved upon LPS challenge.

**Conclusions:**

Our study indicates that obesity-induced downregulation of miR-192 expression exacerbates LPS-induced ALI by promoting macrophage activation. Targeting macrophages and miR-192 may provide new therapeutic avenues for obesity-associated ALI.

**Graphical Abstract:**

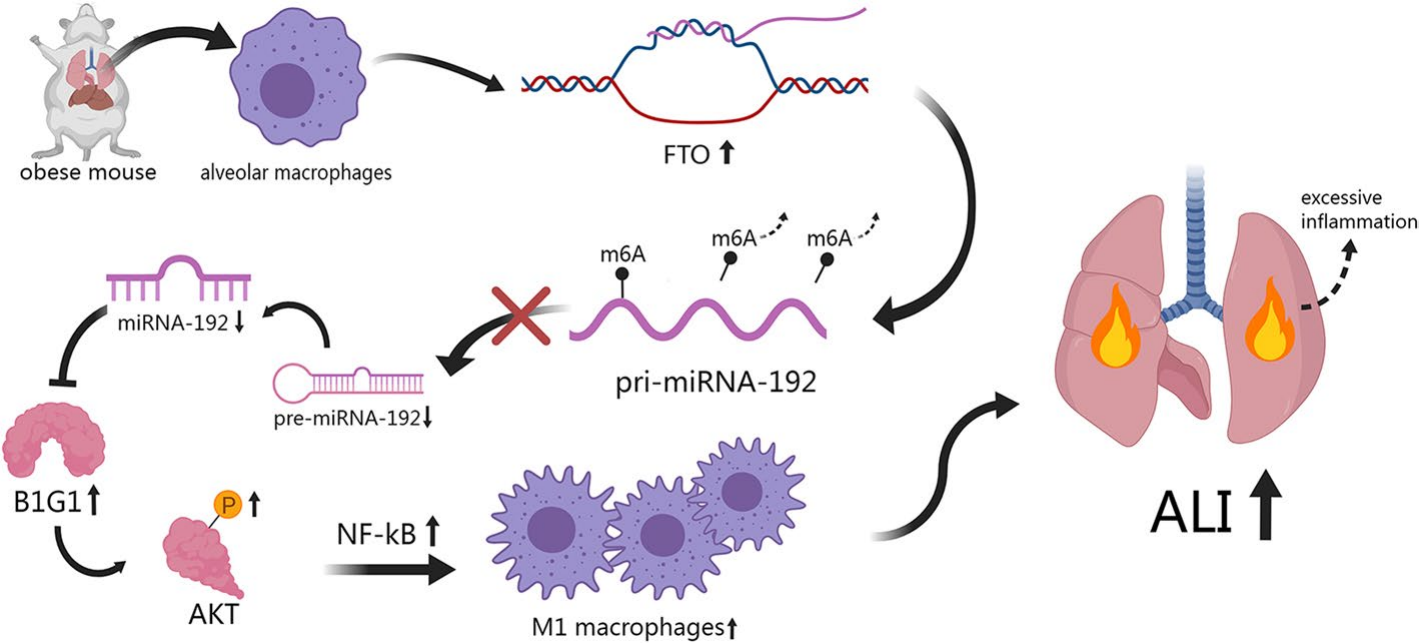

**Supplementary Information:**

The online version contains supplementary material available at 10.1186/s11658-024-00558-w.

## Background

Acute lung injury (ALI) and acute respiratory distress syndrome (ARDS) are inflammatory lung diseases caused by multiple pathogenic factors, with a mortality rate of 35–45% [1]. Obesity is recognized as a significant risk factor for several respiratory diseases, including ARDS, increasing the risk of ARDS and the length of hospital stay [2, 3]. With the rising prevalence of obesity, the number of obese patients with ARDS has significantly increased. Approximately 20% of patients in ICUs are diagnosed as obese [4]. While the influence of obesity on ARDS mortality remains a subject of debate, it undeniably complicates ARDS management. Recent studies, in line with our prior findings, indicate that obesity exacerbates ALI/ARDS induced by Lipopolysaccharide (LPS), particulate matter (PM), and hyperoxia [5–8]. Therefore, understanding the molecular mechanisms through which obesity exacerbates lung injury and developing therapies to halt disease progression are of paramount importance.

Research has revealed that obesity can alter the lung’s inflammatory environment and immune cell populations, subsequently influencing susceptibility to lung injury [9]. Within the lungs, macrophages are the predominant immune cells, and their activation plays a pivotal role in the progression of ALI [10]. Effectively interrupting the lung inflammation cascade and alleviating lung injury can be achieved by inhibiting the pro-inflammatory M1 polarization of macrophage [11]. Increasing evidence suggests that, in the context of obesity, lung tissue macrophages are extensively recruited and polarized towards a pro-inflammatory state, ultimately leading to lung inflammation [9, 12, 13]. Consequently, targeting macrophages presents a promising avenue for new therapeutic strategies to address obesity-related ALI.

MicroRNAs (miRNAs) are endogenous single-stranded non-coding RNAs that play a role in various cellular processes by binding to the 3’ untranslated region (UTR) of target messenger RNAs (mRNAs) to mediate translation repression or degradation [14]. Obesity leads to dysregulation in numerous miRNAs, many of which are implicated in obesity-associated diseases [15, 16]. An increasing body of evidence indicates that miRNAs are intricately linked to the pathogenesis of various inflammatory diseases, offering novel therapeutic targets [14, 17]. However, to date, no studies have explored the role of miRNAs in obesity-related ALI. MiR-192, a member of the miR-192/215 family, plays a pivotal role in immune regulation and inflammation [18]. For example, intra-articular injection of miR-192 agomir alleviates gouty arthritis in mice by inhibiting M1 macrophage polarization [19]. Wang et al. found that baicalin upregulates miR-192 expression, thereby inhibiting the TXNIP/NLRP3/Caspase-1 signaling pathway, ameliorating lipemia-induced pancreatic necrosis and inflammation in rats [20]. In addition, Lou et al. discovered that miR-192 targets MMP-16 and ATG7, thereby alleviating inflammation, airway remodeling, and autophagy in asthmatic mice [21]. Given the critical role of miR-192 in various immune and inflammatory conditions, its potential role in obesity-related ALI/ARDS is worth exploring. However, there are no relevant studies.

In this study, we examined the impact of macrophages on the exacerbation of LPS-induced ALI in the context of obesity. Our results demonstrate that targeting macrophages can ameliorate LPS-induced ALI in obese mice. Furthermore, during obesity, both lung tissue and alveolar macrophages experience downregulation of miR-192, with further suppression following LPS stimulation. In vitro, miR-192 effectively inhibits macrophage activation and inflammation by targeting BIG1. In vivo, overexpression of miR-192 provides protection against ALI in obese mice. Our mechanistic studies reveal that the obesity-induced upregulation of FTO can mediate m6A demethylation, subsequently reducing the expression of miR-192. In summary, our research suggests that miR-192 could serve as a potential therapeutic target for ALI/ARDS in obese individuals.

## Methods

### Human sample collection

Human sample collection was approved by the Ethics Committee of the First Affiliated Hospital of Chongqing Medical University (Approval No: 2023-326). Written informed consent was obtained from all subjects included in this study. Human lung tissue samples were obtained from patients undergoing lung resection surgery for diagnostic and/or therapeutic purposes due to isolated lung nodules or lung tumors at the First Affiliated Hospital of Chongqing Medical University. All subjects were classified into obese (BMI ≥ 30.0 kg/m^2^) and normal weight (BMI 18.5–24.9 kg/m^2^) groups according to the World Health Organization (WHO) BMI standards (Additional file 1: Table S1). All lung tissue samples were meticulously collected at a safe distance of 5 cm from the nodule, strictly ensuring the exclusion of tumor-affected areas. Exclusion criteria included any underlying lung disease, previous chemotherapy or radiotherapy, significant health complications such as cancer or heart disease, pregnancy, or the presence of any other known acute or chronic inflammation.

### Animal studies

Male C57BL/6 mice, aged 6–8 weeks old, provided by the Experimental Animal Center of Chongqing Medical University were used in this study. The animal experiment protocol was approved by the Animal Ethics Committee of Chongqing Medical University (IACUC-CQMU-2023-0062). During the study, mice (5 per cage) were housed in a dedicated pathogen-free facility with temperature control (24 ± 1 °C) and a 12-h light/dark cycle. All mice were randomly assigned to either the normal chow group (Lean group) or a high-fat diet with 60% kcal fat (TP2330055A, Trophic Animal Feed High-tech Co., Ltd., China) group [diet-induced obese (DIO) group]. After 24 weeks of feeding, the mouse ALI model was established according to the literature [22]. Briefly, after anesthesia with pentobarbital (50 mg/kg) (Sigma-Aldrich), 50 μL LPS (Sigma-Aldrich, 5 mg/kg) or saline was instilled intratracheally. To deplete pulmonary macrophages, 50ul of clodronate liposomes (YEASEN, Shanghai, China) or its control liposomes were instilled intratracheally in obese mice 24 h before LPS modeling [23]. To overexpress lung miR-192 levels in mice, miR-192 agomir (10 nmol in a 50 µl volume per mouse) or its control was administered 24 h before LPS injection. 24 h after LPS administration, bronchoalveolar lavage fluid (BALF) or lung tissue was carefully collected for subsequent analysis.

### Cell culture and treatment

The mouse alveolar macrophage cell line (MH-S) was purchased from ATCC and maintained in RPMI-1640 medium (Gibco) supplemented with 10% fetal bovine serum and 1% penicillin–streptomycin, and cultured in a humidified incubator at 37 °C with 5% CO_2_. To mimic the in vivo characteristics of HFD exposure, cells were treated with 100 μM sodium palmitate (PA) or solvent control (Kunchuang, Xian, China) [24, 25]. After 24 h of PA treatment, MH-S cells were stimulated with 100 ng/ml LPS (Sigma, USA) for an additional 24 h.

### Cell transfection and construction of stable cell lines

According to the manufacturer’s instructions, MH-S cells were transfected with a final concentration of 30 nM miR-192 agomir or antagomir and their respective controls using GP-transfect-Mate reagent (GenePharma). Cells were harvested 24 h after transfection for subsequent studies. Lentiviral constructs for BIG1 or FTO overexpression were purchased from Genechem (Shanghai, China). MH-S cells were seeded in a 6-well plate and infected with lentiviral vectors (MOI = 50) in the presence of HitransG A infection enhancer (GeneChem, Shanghai, China) for 12 h. After replacement with complete medium, the cells were further cultured for 60 h. At 72 h post-transfection, stable transductions were selected with puromycin (3 μg/ml) (Meilunbio, MA0318). Agomir and antagomir sequences are listed in Additional file 1: Table S2.

### Collection of bronchoalveolar lavage fluid and extraction of alveolar macrophages

Mice were euthanized, and the trachea was exposed. An 18-gauge catheter was inserted into the trachea through a midline incision extending from the upper abdomen to the mid-neck region. The lungs were lavaged with 1.2 ml of cold PBS containing 0.5 mM EDTA. After 1 min, the lavage fluid was gently aspirated and collected in a 15 ml centrifuge tube. This procedure was repeated 10 times to obtain bronchoalveolar lavage fluid (BALF). The centrifuge tube was then spun at 400 × g at 4 °C for 10 min. The supernatant was used for total protein assay, and the cell pellet was resuspended in RPMI-1640 medium supplemented with 10% FBS and seeded in a 6-well plate. Alveolar macrophages were allowed to adhere for 2.5 h, after which non-adherent cells were washed away with PBS [26]. Lung tissue after alveolar lavage was not used for follow-up studies.

### Hematoxylin and Eosin (H&E) staining

The lower lobe of the right lung was fixed in 4% paraformaldehyde (Servicebio, G1101) for 24 h, followed by paraffin embedding, and sectioning at 5 μm. Tissue sections were stained according to the standard H&E staining protocol. The pathological scoring was determined as previously described [27].

### Biochemical analysis

Lung LDH activity was measured using a commercial kit (Solarbio, Beijing, China). Levels of pro-inflammatory cytokines TNF-α, IL-1β and IL-6 in lung tissue and cell culture medium were determined using commercial ELISA kits (MEIKE Jiangsu Sumeike Biological Technology Co., Ltd, Shanghai, China).

### Lung wet/dry (W/D) weight ratio

The right apical lung tissue was weighed to record its wet weight (W). The tissue was then dehydrated at 80 °C for 48 h and weighed again to determine its dry weight (D).

### Immunofluorescence

After dewaxing and rehydration, paraffin sections of lung tissue were subjected to antigen retrieval using citrate sodium antigen retrieval solution (Beyotime, P0081). After fixation in 4% paraformaldehyde, MH-S cells were permeabilized with 0.1% Triton X-100 (Beyotime, ST797). Tissues / cells were incubated with 10% fetal bovine serum (Beyotime, C0234) for 1 h at room temperature to block non-specific antigens. The slides were incubated with the primary antibody overnight at 4 °C, followed by incubation with fluorescence-conjugated secondary antibodies (Proteintech, Wuhan, China) for 1 h at room temperature. Sections were mounted with anti-fade mounting medium containing DAPI (Beyotime, P0131). Tissue fluorescence was captured using an upright fluorescence microscope (Nikon, Tokyo, Japan), while cell fluorescence was captured under a confocal microscope (ZEISS, Oberkochen, Germany). Immunofluorescence intensity and the number of positive cells were determined using FlowJo software (version 10, FlowJo, LLC). Primary antibodies used included anti-F4/80 (Thermo Fisher Scientific, Waltham, MA, USA), anti-FTO (Abcam, Cambridge, UK) and anti- phospho-NF-κB p65 (Cell Signaling Technology, MA, USA).

### RT-PCR

Total RNA was extracted from lung tissue and cells using the TRIzol reagent (Takara Biotechnology) according to the manufacturer’s instructions, and then reverse transcribed into cDNA using the Evo M-MLV RT Mix Kit (Accurate Biology, AG11728) or miRNA 1st strand cDNA synthesis kit (Accurate Biology, AG11716). Subsequently, qPCR was performed using the SYBR Green Premix Pro Taq HS qPCR Kit II (Accurate Biology, AG11702). The relative expression levels of miRNAs or mRNAs were normalized to U6 small nuclear RNA (snRNA) or β-actin using the 2-ΔΔCt comparison method. Primers used in this study are listed in Additional file 1: Table S3.

### Dual-luciferase reporter assay

Potential target genes and their binding sites for miR-192 were predicted using the TargetScan, miRDB and miRWalk databases. The ARFGEF1-3 ‘UTR wild-type (WT) and mutant (MUT) reporter vectors were constructed by Jima Company, Shanghai, China. MH-S cells were cultured in 12-well plates and co-transfected with luciferase vectors and miR-192 agomir or its control (NC) using the GP-transfect-Mate reagent (GenePharma). Cells were lysed 48 h post-transfection and luciferase activity was measured using the dual-luciferase reporter assay system (Promega, San Luis Obispo, CA, USA). Results were expressed as the ratio of firefly luciferase to Renilla luciferase luminescence intensity.

### MiR-192 activity assay

To assess changes in miR-192 activity after a high-fat diet (HFD), we used the R software (version 4.3.1, R Foundation for Statistical Computing, Vienna, Austria) and its ggplot2 and dplyr packages for our analyses. First, we performed microarray analysis of mouse lung lobes on normal diet or HFD diet for 12 weeks. RNA labelling and microarray hybridisation were performed by Kangchen Biotechnology Ltd. according to Agilent Monochrome Microarray Gene Expression Analysis protocol. Samples were then amplified and transcribed into fluorescent cRNA using a random priming method, followed by purification, hybridisation and scanning on an Agilent DNA microarray scanner. Microarray probe signals were acquired using Agilent Feature Extraction software and data were normalised using Agilent GeneSpring GX v12.1 software. Using these data, we calculated the log2 fold change (log2FC) for each gene between HFD and control. Based on the list of target genes of miR-192 predicted from the three databases above, we further categorised the genes as either target or non-target genes of miR-192. the Kolmogorov–Smirnov (KS) test was used to compare the log2FC distributions of the genes in these two groups. Distribution differences were visualised by cumulative distribution function plots. The microarray data have been uploaded to the NCBI Gene Expression Omnibus (GEO) under accession number GSE 229262.

### m6A quantification

Total RNA was extracted from lung tissues using the TRIzol reagent. The relative m6A content was measured using the EpiQuik m6A RNA Methylation Quantification Kit (colorimetric) (Epigentek, P-9005) according to the manufacturer’s instructions. The percentage of m6A in total RNA can be calculated using the formula: m6A% = [(sample OD − NC OD)/S]/ [(PC OD − NC OD)/P] × 100%, where NC (negative control) represents RNA without m6A, PC (positive control) represents m6A oligonucleotides, normalized to 100% m6A, S is the amount of sample RNA input, and P is the amount of positive control input.

### RNA-binding protein immunoprecipitation (RIP)

RIP assays were performed according to the manufacturer’s instructions using the Magna RIP™ RNA-Binding Protein Immunoprecipitation Kit (Millipore, Cat. No. 17-700). Briefly, single cell suspensions of lung tissue and MH-S cells were prepared in ice-cold PBS and lysed in RIP lysis buffer on ice for 5 min. After centrifugation, the supernatant was incubated overnight at 4 °C with magnetic beads conjugated to either anti-m6A antibody (Abcam, ab151230) or control immunoglobulin G (IgG, Millipore, USA). RNA from the immunoprecipitates was then extracted with TRIzol and subjected to qRT-PCR using primers specific for pri-miR-192.

### Flow cytometry analysis

Cells were scraped from the culture dish and then washed three times with cold PBS. Staining was performed according to the manufacturer’s recommended protocol using PE anti-mouse CD86 (12-0862-82, Thermo Fisher, USA) and APC anti-mouse CD206 (17–2061-82, Thermo Fisher, USA) antibodies. Data acquisition and further analysis was conducted using the CytExpert software (version 10.0.7; Tree Star, Ashland, OR, USA).

### Western blot

Lung tissues and cells were collected, and proteins were extracted using RIPA lysis buffer (Beyotime, China). Protein concentration was determined using the BCA method (Beyotime, China). Proteins were separated on SDS-PAGE gels and transferred to polyvinylidene fluoride (PVDF) membranes (Millipore, Billerica, MA, USA), which were blocked with 5% nonfat milk for 1 h at room temperature. Immunoblotting was carried out at 4 °C overnight using primary antibodies directed against BIG1 (1:1000, Abcam, ab183747), FTO (1:1000, Abcam, ab126605), METTL3 (1:1000, Abcam, ab195352), ALKBH5 (1:1000, Abcam, ab195377), YTHDC2 (1:1000, Abcam, ab220160), phospho-AKT (1:1000, Cell Signaling Technology, 4060T), AKT1 (1:1000, Cell Signaling Technology, 2938T), phospho-NF-κB p65 (1:1000, Cell Signaling Technology, 3033T), NF-κB (1:1000, Cell Signaling Technology, 8242), and β-actin (1:2000, Proteintech, 20536-1-AP). After washing three times, the membranes were incubated with secondary antibodies (SA00001-2, Proteintech) for 1 h at room temperature. The relative intensities of the protein bands were analyzed using the Bio-Rad Quantity One software. Results for phospho-AKT and phospho-NF-κB p65 were normalized to their respective total protein levels (AKT1 and NF-κB), while other protein levels were normalized to β-actin.

### Statistical analysis

All data are expressed as the mean ± SD. Statistical analyses were conducted using the GraphPad Prism 9.5 software (USA). The Student’s t-test was used for two group comparisons, and one-way ANOVA was employed for multiple group comparisons. In all analyses, statistical significance was set at *P* < 0.05.

## Results

### Macrophages contribute to the exacerbation of LPS-induced ALI in obesity

**Fig. 1:**
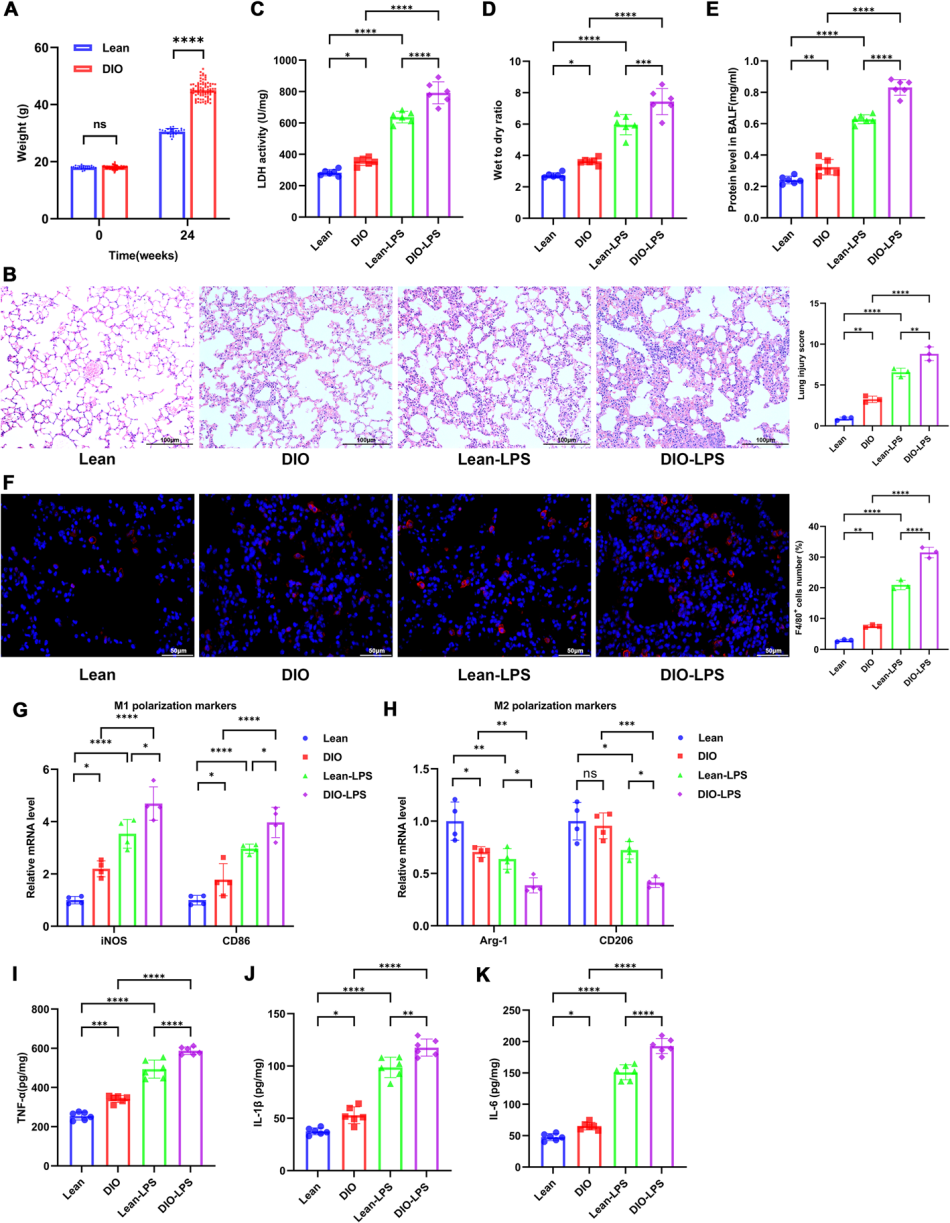
Obesity aggravates LPS-induced pulmonary macrophage activation and ALI. **A** Body weight of Lean and DIO mice during normal chow and high-fat diet feeding, respectively. *n* = 24 in the Lean mice group and *n* = 80 in the DIO mice group. **B** H&E staining illustrating the extent of lung pathological damage and corresponding lung injury scores. **C** LDH activity in lung tissue was assessed using an LDH activity assay kit. **D** Wet-to-dry weight ratio of the lungs. **E** Protein concentration in the BALF supernatant was determined using the BCA protein quantitation kit. **F** Immunofluorescent labeling of macrophages. Macrophages are labeled in red (F4/80) and nuclei in blue (DAPI). F4/80-positive cells were counted using FLOWJO software. Scale bar, 50 µm. **G**, **H** RT-qPCR assessment of mRNA levels of M1 macrophage markers (iNOS and CD86) and M2 macrophage markers (Arg-1 and CD206) in lung tissues. **I**–**K** ELISA measurements of protein levels of TNF-α, IL-1β, and IL-6 in lung tissues. Data are expressed as the mean ± SD, *P < 0.05. **P < 0.01. ***P < 0.001. ****P < 0.0001

Compared to mice fed a regular diet (Lean group), the weight of mice fed a high-fat diet (DIO group) increased significantly (Fig. 1A). Hematoxylin and eosin (H&E) staining revealed that, in comparison to Lean mice, DIO mice exposed to LPS demonstrated more severe pathological damages, such as inflammatory cell infiltration, alveolar wall thickening, and alveolar collapse (Fig. 1B). Consistent with the pathological findings, LDH activity in lung tissue, wet/dry ratio (W/D), and protein concentration in BALF were notably increased in DIO-LPS mice (Fig. 1C–E), indicating that LPS-induced ALI is exacerbated during obesity. Macrophages play a pivotal role in the progression of ALI [28], yet their role in the exacerbation of ALI during obesity remains unreported. Immunofluorescence results showed that, regardless of LPS treatment, the number of pulmonary macrophages (F4/80^+^) significantly increased in obesity (Fig. 1F). Macrophages primarily activate into pro-inflammatory M1 type and anti-inflammatory M2 type [10]. RT-qPCR findings revealed that the mRNA levels of M1 macrophage markers, iNOS and CD86, increased significantly during obesity, while the levels of M2 macrophage markers, Arg-1 and CD206, showed an opposite trend. Moreover, these expressions were further intensified post-LPS stimulation (Fig. 1G, H). ELISA results demonstrated that the expression of pro-inflammatory cytokines (TNF-α, IL-1β, and IL-6) in lung tissue also significantly increased (Fig. 1I–K).

**Fig. 2:**
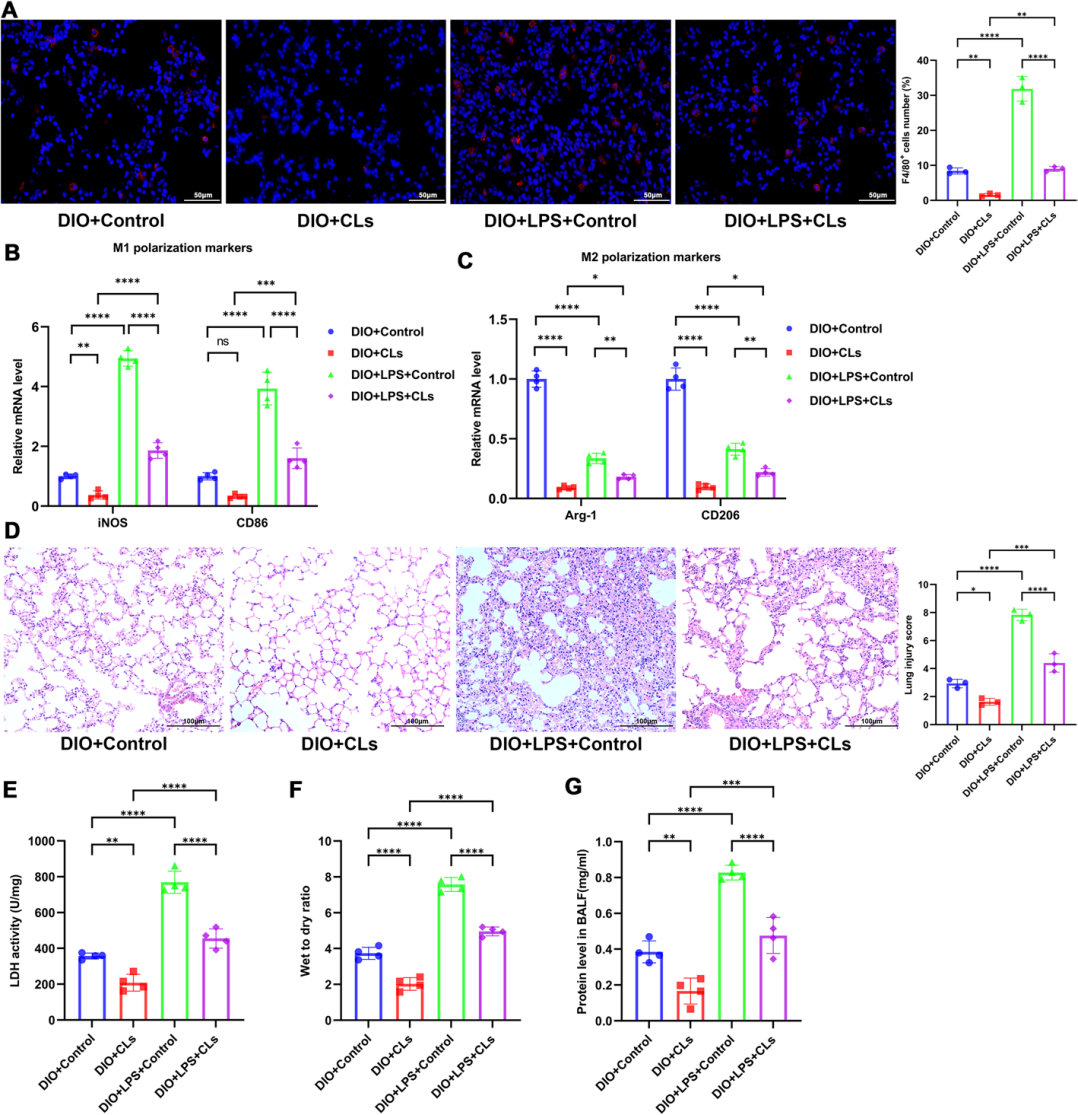
Macrophage depletion alleviates LPS-induced ALI in obese mice. Macrophages in lung tissue of obese mice were depleted through intratracheal instillation of clodronate liposomes (CLs). **A** Immunofluorescent labeling of macrophages. Macrophages are labeled in red (F4/80) and nuclei in blue (DAPI). F4/80-positive cells were counted using FLOWJO software. Scale bar, 50 µm. **B**, **C** RT-qPCR assessment of mRNA levels of M1 macrophage markers (iNOS and CD86) and M2 macrophage markers (Arg-1 and CD206) in lung tissues. **D** H&E staining illustrating the extent of lung pathological damage and corresponding lung injury scores. **E** LDH activity in lung tissue was assessed using an LDH activity assay kit. **F** Wet-to-dry weight ratio of the lungs. **G** Protein concentration in the BALF supernatant was determined using the BCA protein quantitation kit. Data are expressed as the mean ± SD, *P < 0.05. **P < 0.01. ***P < 0.001. ****P < 0.0001

To further explore the role of macrophages in the exacerbation of obesity-associated ALI, we depleted macrophages in the lung tissue of obese mice by intratracheal instillation of clodronate liposomes (CLs) [29]. Immunofluorescence results showed that the number of macrophages (F4/80^+^) in the lung tissue significantly decreased 24 h post CLs administration (Fig. 2A). Furthermore, the mRNA levels of both M1 and M2 macrophage markers significantly decreased (Fig. 2B, C). HE staining demonstrated that, regardless of LPS exposure, the pathological damage in the lungs of obese mice was significantly ameliorated after macrophage depletion (Fig. 2D). LDH activity in lung tissue, W/D and protein concentration in BALF were all significantly reduced post macrophage depletion (Fig. 2E–G). In summary, our results suggest that obesity exacerbates LPS-induced lung injury and macrophage activation in mice, with macrophages playing a key role in obesity-exacerbated LPS-induced ALI.

### The expressions of miR-192 in lung tissue and alveolar macrophages are reduced during obesity and are further decreased with LPS stimulation

**Fig. 3:**
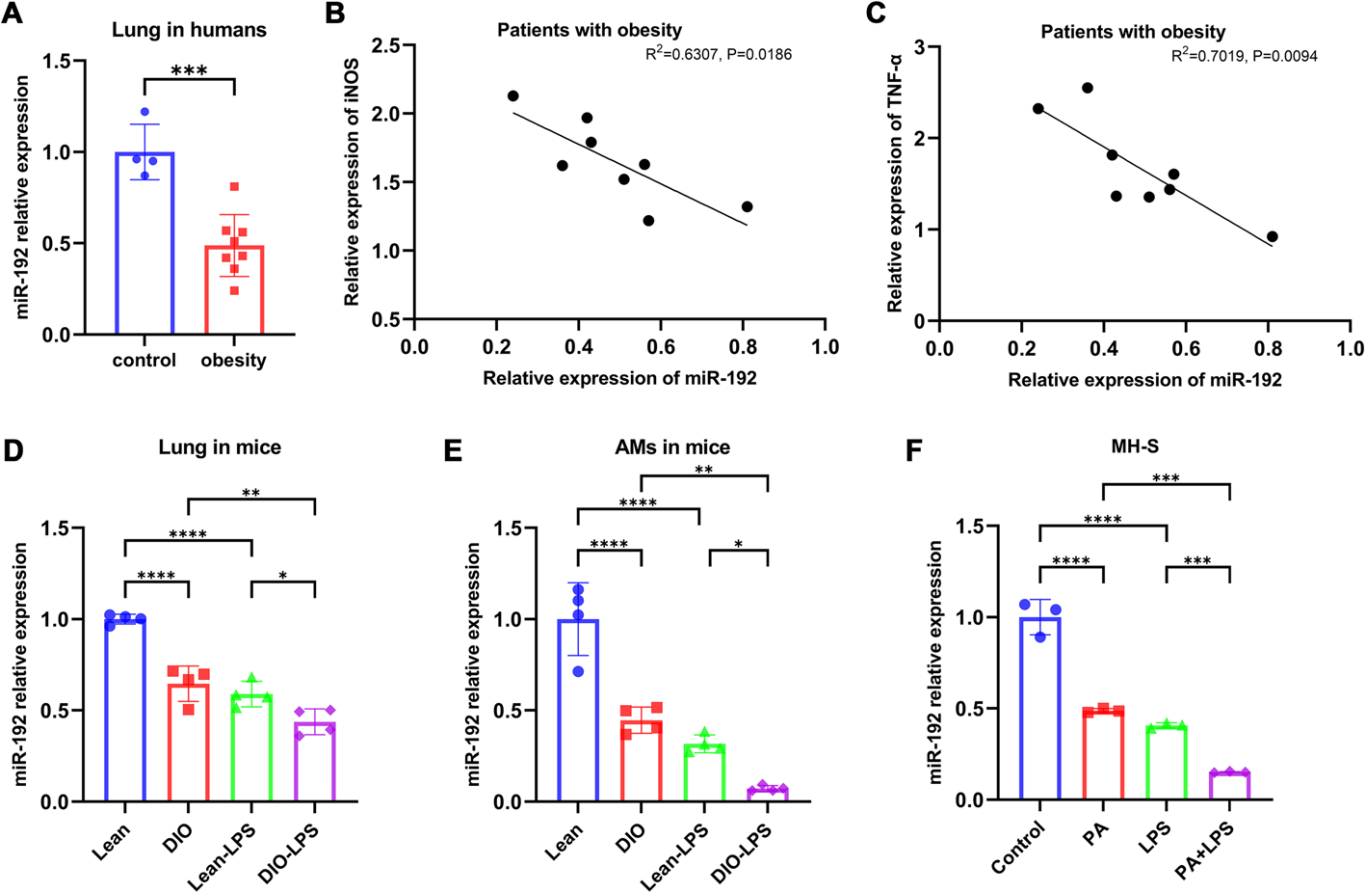
Downregulation of miR-192 in lung tissues and alveolar macrophages during obesity, further decreased following LPS stimulation. **A** RT-qPCR assessment of miR-192 levels in lung tissues of 8 obese and 4 control patients. **B** Pearson correlation between the relative expression of miR-192 and iNOS in lung tissues of obese patients (R^2^ = 0.6307, *P* = 0.0186). **C** Pearson correlation between the relative expression of miR-192 and TNF-α in lung tissues of obese patients (R^2^ = 0.7019, *P* = 0.0094). **D**, **E** RT-qPCR assessment of miR-192 levels in mouse lung tissues and AMs. **F** RT-qPCR assessment of miR-192 levels in MH-S cells. Data are expressed as the mean ± SD, *P < 0.05. **P < 0.01. ***P < 0.001. ****P < 0.0001

We measured the expression levels of miR-192 in lung tissues of obese (*n* = 8) and control (*n* = 4) patients using RT-qPCR. The expression of miR-192 in obese patients was significantly lower than that in the control group (Fig. 3A). Moreover, we observed a negative correlation between miR-192 expression and the levels of M1 macrophage activation markers iNOS and TNF-α in lung tissues of obese patients (Fig. 3B, C). We further examined the expression of miR-192 in mice fed with a high-fat diet. Similar to human tissues, the expression of miR-192 was significantly reduced in lung tissues of obese mice and further decreased following LPS stimulation (Fig. 3D). Quantitative analysis of fold changes in all gene mRNA levels in lung tissues from mice fed with high-fat or normal diet for 12 weeks showed significant upregulation of mRNAs carrying the miR-192 binding site (miR-192 targets), indicating reduced miR-192 activity in HFD (Additional file 1: Fig S1A).

To delve deeper into the impact of obesity and metabolic stress on miR-192 expression in alveolar macrophages, we assessed the expression of miR-192 in freshly isolated mouse alveolar macrophages (AMs) and palmitic acid (PA) treated MH-S cells. In comparison to lean mice, the expression of miR-192 in DIO mouse AMs was significantly reduced and further decreased post LPS stimulation (Fig. 3E). PA, the most abundant saturated fatty acid, is commonly utilized in vitro to mimic the metabolic stress state of obesity [24, 25]. Following PA treatment, miR-192 expression in MH-S cells was reduced and further diminished after LPS stimulation (Fig. 3F).

### Increased m6A demethylation during obesity mediates the downregulation of miR-192 expression

**Fig. 4:**
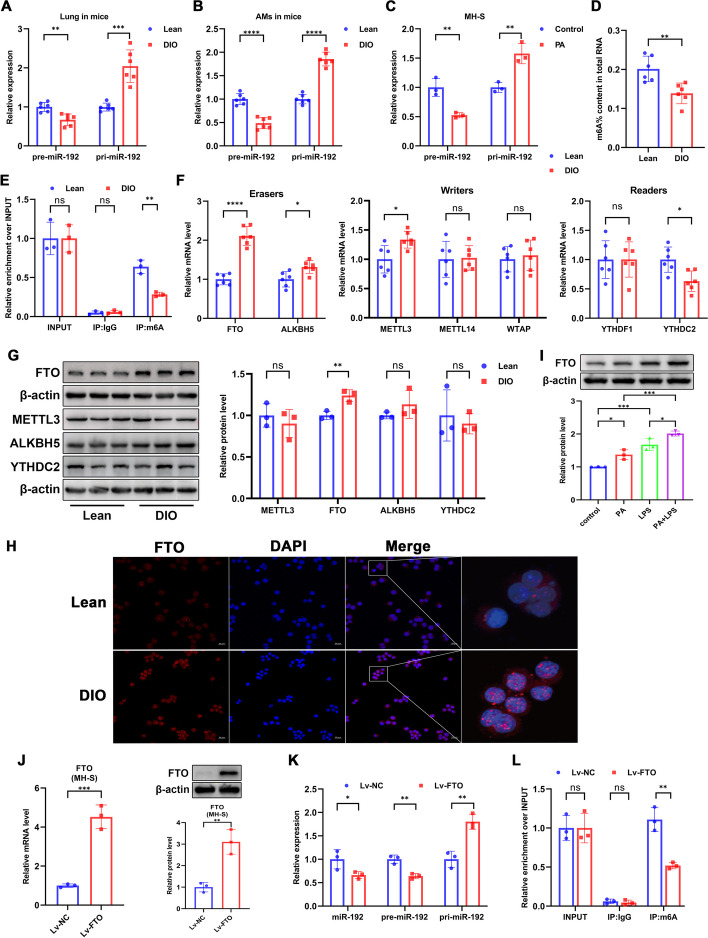
Increased m6A demethylation during obesity mediates the downregulation of miR-192 expression. **A**, **B** RT-qPCR assessment of pre-miR-192 and pri-miR-192 expression in mouse lung tissues and AMs. **C** RT-qPCR assessment of pre-miR-192 and pri-miR-192 expression in PA-treated MH-S cells. **D** m6A levels comparison in lung tissues of lean vs. obese mice. **E** m6A RIP assessment of m6A-modified pri-miR-192 expression in lung tissues. **F** RT-qPCR assessment of mRNA levels of METTL3, METTL14, WTAP, FTO, ALKBH5, YTHDF1, and YTHDC2. **G** Western blot assessment of protein expression of METTL3, FTO, ALKBH5, and YTHDC2 in lung tissues. **H** Immunofluorescence of FTO (red) in freshly extracted AMs with nuclei labeled in blue (DAPI). Scale bar, 20 µm. **I** Western blot assessment of FTO protein expression in MH-S cells after PA or/and LPS treatment. **J** RT-qPCR and western blot assessment of FTO mRNA and protein expression in MH-S cells after Lv-FTO transfection. **K** RT-qPCR assessment of miR-192, pre-miR-192 and pri-miR-192 expression in MH-S cells after Lv-FTO transfection. **L** m6A RIP assessment of m6A-modified pri-miR-192 expression in MH-S cells after Lv-FTO transfection. Data are expressed as the mean ± SD, *P < 0.05. **P < 0.01. ***P < 0.001. ****P < 0.0001

To investigate the cause of miR-192 downregulation during obesity, we examined the expression of pre-miR-192 (precursor miR-192) and pri-miR-192 (miR-192 transcript) in lung tissue. RT-qPCR results indicated that while pre-miR-192 decreased in lung tissues of obese mice, pri-miR-192 actually increased (Fig. 4A). A similar trend was observed in freshly extracted AMs and in MH-S cells treated with PA (Fig. 4B, C), suggesting that the downregulation of miR-192 might be related to the obstruction of pri-miR-192 processing. Since m6A modification has recently been widely reported to promote pri-miRNA processing [30–32], we assessed the overall m6A methylation level in lung tissues. The m6A methylation level was found to be significantly reduced in obese mouse lung tissues (Fig. 4D). RIP experiments revealed that pri-miR-192 in lung tissues could undergo m6A modification, and this m6A-modified pri-miR-192 decreased during obesity (Fig. 4E).

Subsequently, we evaluated the expression of several m6A-related enzymes in lung tissues. RT-qPCR analyses revealed significant expression differences in METTL3, FTO, ALKBH5, and YTHDC2 in the lung tissues of lean and obese mice (Fig. 4F). Western blot analyses further pinpointed that only FTO, the key enzyme for m6A demethylation, exhibited significant expression changes, showing an increase in obese lung tissues (Fig. 4G). Immunofluorescence studies confirmed that FTO fluorescence intensity was significantly higher in AMs isolated from obese mice compared to lean mice (Fig. 4H). Notably, FTO expression in MH-S cells was significantly upregulated following PA treatment and further elevated after lipopolysaccharide (LPS) stimulation (Fig. 4I). Next, we constructed a stable lentiviral cell line by transfecting MH-S cells with lentivirus overexpressing FTO (Lv-FTO). Lv-FTO significantly increased both mRNA and protein expression of FTO in MH-S cells (Fig. 4J). RT-qPCR results indicated that Lv-FTO significantly decreased the expression of mature miR-192 and pre-miR-192 and promoted the accumulation of pri-miR-192 (Fig. 4K). RIP experiments showed that overexpressing FTO also significantly decreased the level of m6A-modified pri-miR-192 (Fig. 4L). These findings suggest that obesity-induced FTO overexpression inhibits miR-192 production by promoting the m6A demethylation of pri-miR-192.

### miR-192 inhibition exacerbates LPS-induced activation and inflammation of MH-S cells

**Fig. 5:**
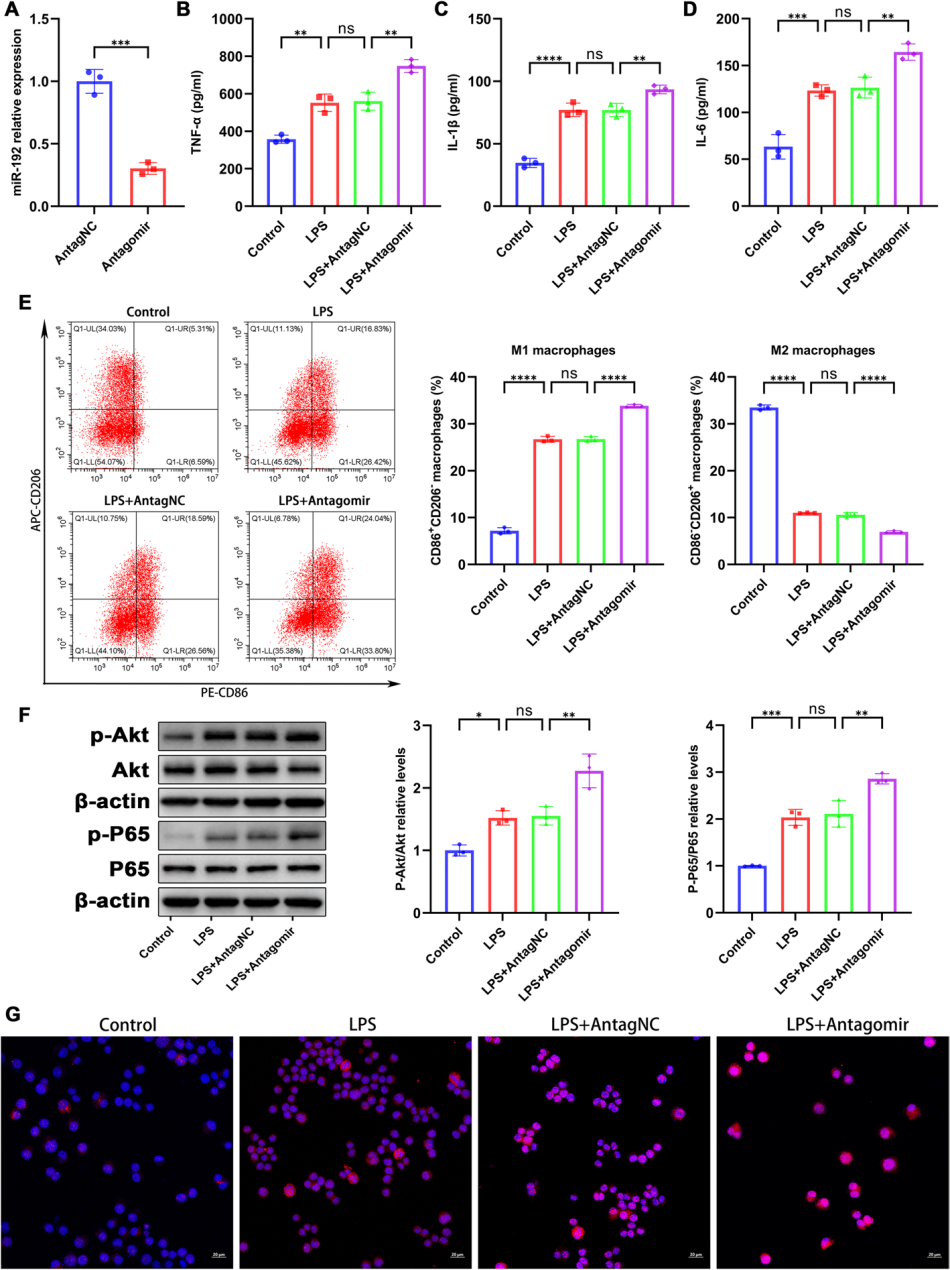
miR-192 inhibition intensifies LPS-induced activation and inflammation in MH-S cells. MH-S cells were treated with the miR-192 inhibitor (Antagomir) or its control (AntagNC). **A** RT-qPCR of miR-192 expression post Antagomir treatment in MH-S cells. **B**–**D** ELISA measurements of protein levels of TNF-α, IL-1β, and IL-6 in the cell culture supernatant. **E** Flow cytometry analysis of the proportions of M1 macrophages (CD86^+^CD206^−^) and M2 macrophages (CD86^−^CD206^+^) in MH-S cells. **F** WB assessment of p-AKT, AKT, p-p65, and p65 levels. **G** Immunofluorescence of nuclear p-p65 translocation. Red for p-p65, blue for DAPI-stained nuclei. Scale bar, 20 μm. Data are expressed as the mean ± SD, **P < 0.01. ***P < 0.001. ****P < 0.0001

To explore the effects of miR-192 downregulation on macrophage activation, MH-S cells were treated with the miR-192 inhibitor (Antagomir) or its control (AntagNC). RT-qPCR confirmed that the Antagomir notably reduced miR-192 expression in MH-S cells (Fig. 5A). ELISA showed that LPS elevated the secretion of pro-inflammatory cytokines (TNF-α, IL-1β, and IL-6) in MH-S cells, and this rise was further accentuated by the Antagomir (Fig. 5B–D). Flow cytometry findings showed that LPS increased the proportion of M1 macrophages (CD86^+^CD206^−^) and reduced the proportion of M2 macrophages (CD86^−^CD206^+^), and inhibition of miR-192 further accentuated the M1 to M2 macrophage ratio (Fig. 5E). Recognizing the AKT/NF-κB pathway as key in macrophage activation [33], we evaluated the related protein expression. Western blot results showed enhanced phosphorylation of AKT and NF-κB p65 post LPS exposure, with this effect further magnified when combined with Antagomir (Fig. 5F). Immunofluorescence revealed that Antagomir notably augmented the nuclear presence of phospho-p65 triggered by LPS, supporting the WB observations (Fig. 5G). Our data indicate that miR-192 inhibition amplifies LPS-driven macrophage activation and inflammation.

### miR-192 overexpression counteracts PA and LPS-induced activation and inflammation of MH-S cells

**Fig. 6:**
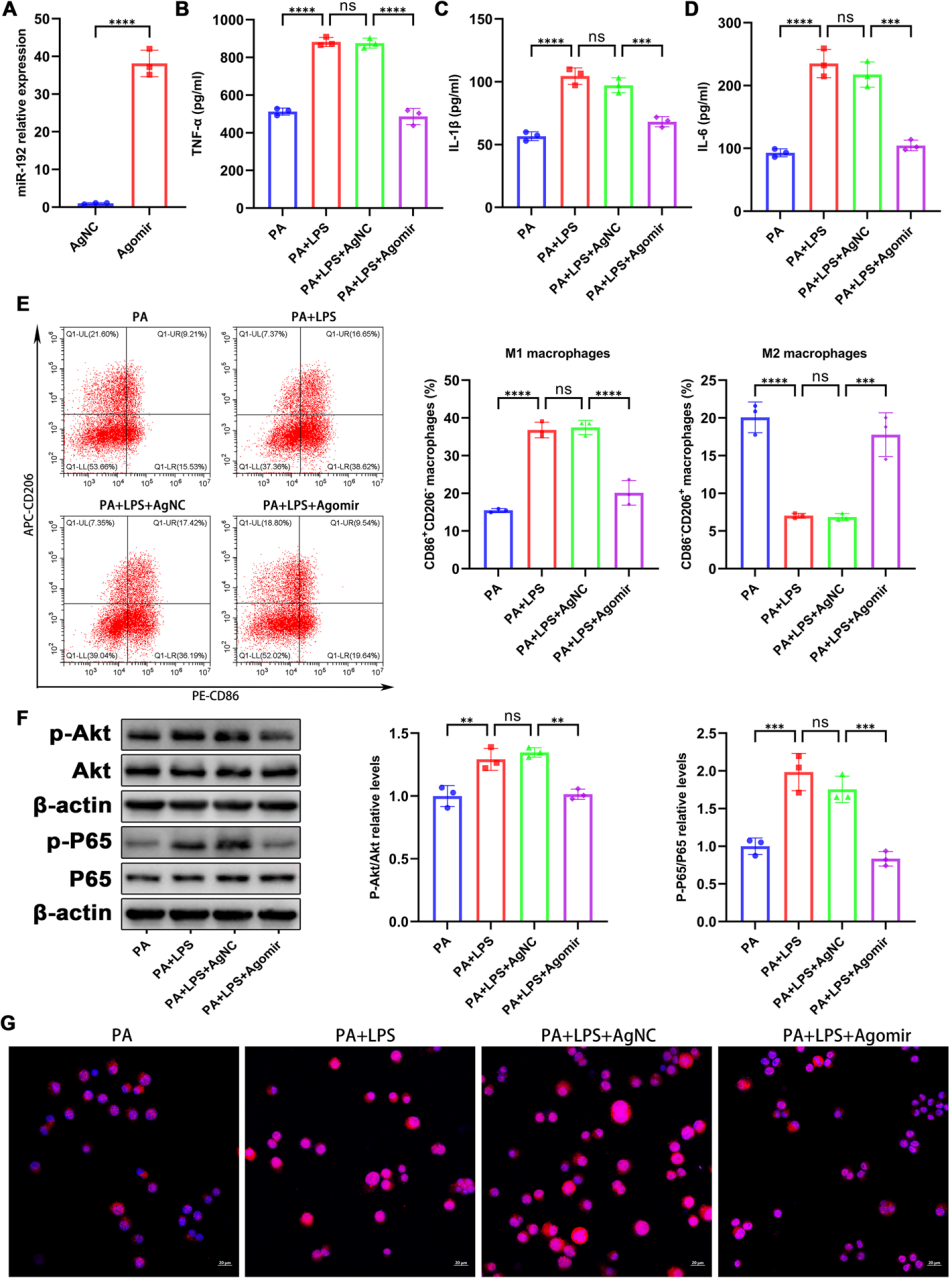
miR-192 overexpression counteracts PA and LPS-induced activation and inflammation of MH-S cells. MH-S cells were treated with the miR-192 activator (Agomir) or its control (AgNC). **A** RT-qPCR of miR-192 expression post Agomir treatment in MH-S cells. **B**–**D** ELISA measurements of protein levels of TNF-α, IL-1β, and IL-6 in the cell culture supernatant. **E** Flow cytometry analysis of the proportions of M1 macrophages (CD86^+^CD206^−^) and M2 macrophages (CD86^−^CD206^+^) in MH-S cells. **F** WB assessment of p- AKT, AKT, p-p65, and p65 levels. **G** Immunofluorescence of nuclear p-p65 translocation. Red for p-p65, blue for DAPI-stained nuclei. Scale bar, 20 μm. Data are expressed as the mean ± SD, **P < 0.01. ***P < 0.001. ****P < 0.0001

To investigate the potential therapeutic effects of miR-192 upregulation on macrophage activation, MH-S cells were treated with a miR-192 activator (Agomir) or its control (AgNC). RT-qPCR demonstrated that the Agomir notably elevated miR-192 expression in MH-S cells (Fig. 6A). ELISA analysis showed that the combined treatment of PA and LPS boosted the secretion of pro-inflammatory cytokines (TNF-α, IL-1β, and IL-6) in MH-S cells, an effect which was mitigated by the Agomir treatment (Fig. 6B–D). Flow cytometry results indicated that the combined PA and LPS treatment favored the M1 macrophage phenotype (CD86^+^CD206^−^) while reducing the M2 phenotype (CD86^−^CD206^+^); these shifts were notably reversed with the introduction of miR-192 Agomir (Fig. 6E). Expanding on the AKT/NF-κB pathway, western blot findings revealed that PA combined with LPS stimulation heightened the phosphorylation levels of AKT and NF-κB p65, while miR-192 overexpression significantly toned down these elevations (Fig. 6F). Immunofluorescence results highlighted that Agomir significantly tempered the nuclear translocation of phospho-p65 instigated by the PA and LPS combination, in line with the WB findings (Fig. 6G). Collectively, our observations suggest that miR-192 overexpression counteracts the macrophage activation and inflammation triggered by PA and LPS.

### miR-192 targets BIG1 in alveolar macrophages

**Fig. 7:**
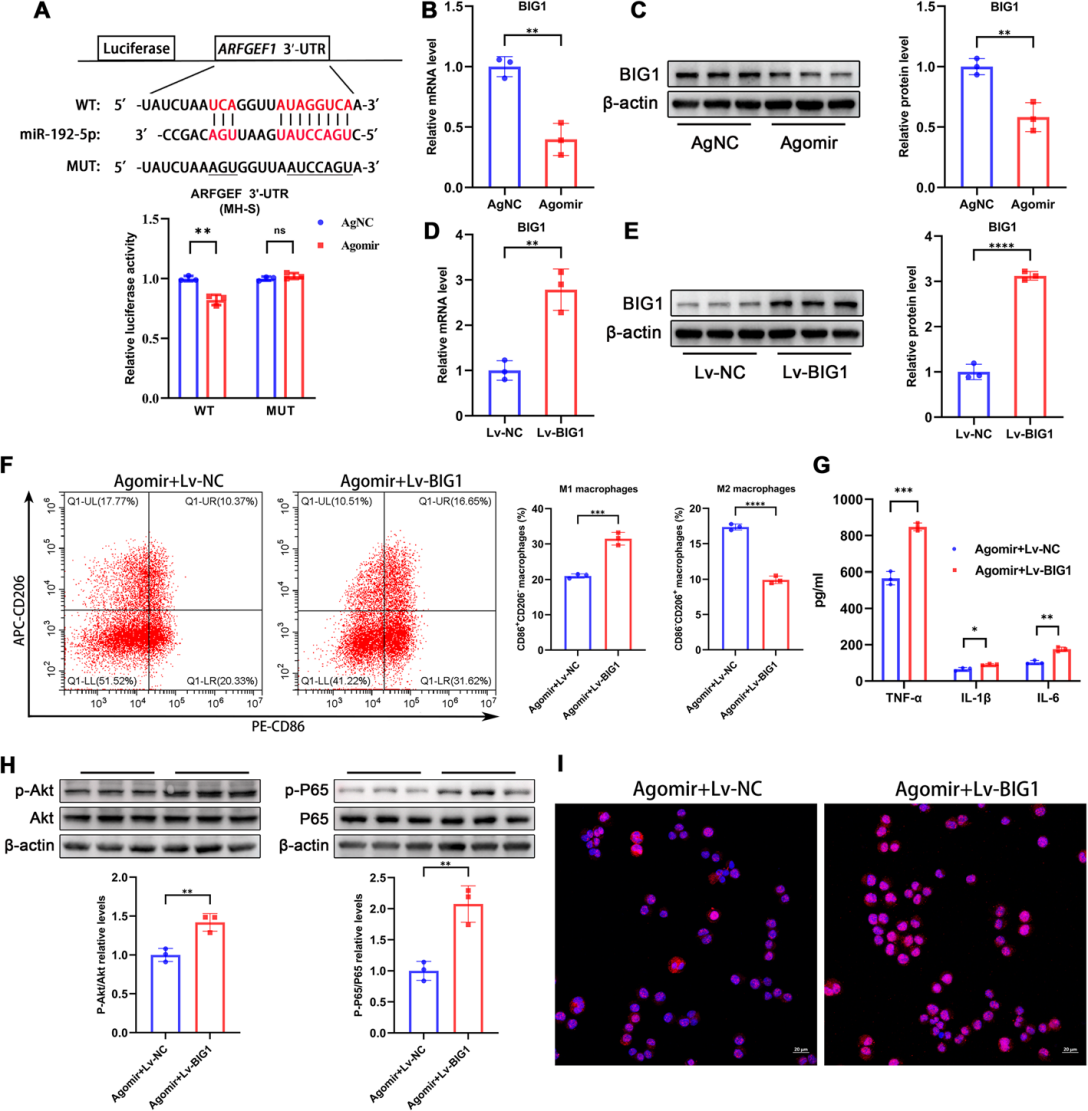
miR-192 targets BIG1 in MH-S cells. **A** Bioinformatics prediction of the binding site between miR-192 and ARFGEF1 (BIG1) 3ʹ UTR, validated by dual-luciferase reporter assay. **B**, **C** RT-qPCR and western blot assessment of BIG1 mRNA and protein expression in MH-S cells after Agomir transfection. **D**, **E** RT-qPCR and western blot assessment of BIG1 mRNA and protein expression in MH-S cells after Lv-BIG1 transfection. **F** Flow cytometry analysis of the proportions of M1 macrophages (CD86^+^CD206^−^) and M2 macrophages (CD86^−^CD206^+^) in MH-S cells. **G** ELISA measurements of protein levels of TNF-α, IL-1β, and IL-6 in the cell culture supernatant. **H** WB assessment of p- AKT, AKT, p-p65, and p65 levels. **I** Immunofluorescence of nuclear p-p65 translocation. Red for p-p65, blue for DAPI-stained nuclei. Scale bar, 20 μm. Data are expressed as the mean ± SD, *P < 0.05. **P < 0.01. ***P < 0.001. ****P < 0.0001

To delve deeper into the downstream molecular mechanisms of miR-192, we predicted its target genes using bioinformatics. Among them, ARFGEF1 (BIG1) was consistently predicted in multiple databases, and the binding sites between it and miR-192 were conserved across species (Additional file 1: Fig S2A). BIG1 has been proven to be closely related to macrophage activation [34, 35]. To provide direct evidence that BIG1 is a target of miR-192, we conducted a dual-luciferase reporter assay in MH-S cells. The results demonstrated that miR-192 significantly reduced the luciferase activity of the wild-type 3′ UTR of BIG1, but it had no inhibitory effect on the mutated 3′ UTR of BIG1 (Fig. 7A). Moreover, we observed a significant decrease in both mRNA and protein expression levels of BIG1 in MH-S cells upon miR-192 overexpression (Fig. 7B, C).

To further investigate the role of BIG1 in the miR-192-mediated suppression of macrophage activation, MH-S cells were co-treated with Lv-BIG1 or Lv-NC and Agomir prior to stimulation with PA combined with LPS. We found that Lv-BIG1 significantly upregulated mRNA and protein expression of BIG1 in MH-S cells (Fig. 7D, E). Flow cytometry results revealed that Lv-BIG1 could offset the suppressive effect of miR-192 Agomir on the M1 to M2 macrophage ratio (Fig. 7F). ELISA results indicated that Lv-BIG1 also significantly counteracted the inhibitory effect of miR-192 Agomir on inflammatory cytokines (Fig. 7G). WB results showed increased phosphorylation levels of p-AKT and p65 when co-treated with Lv-BIG1 and miR-192 Agomir (Fig. 7H). Immunofluorescence analysis indicated an enhanced nuclear translocation of p65 (Fig. 7I). In summary, our findings suggest that miR-192 suppresses macrophage activation and inflammation induced by PA combined with LPS by targeting BIG1.

### Overexpression of miR-192 protects obese mice from LPS-induced pulmonary macrophage activation and ALI

**Fig. 8:**
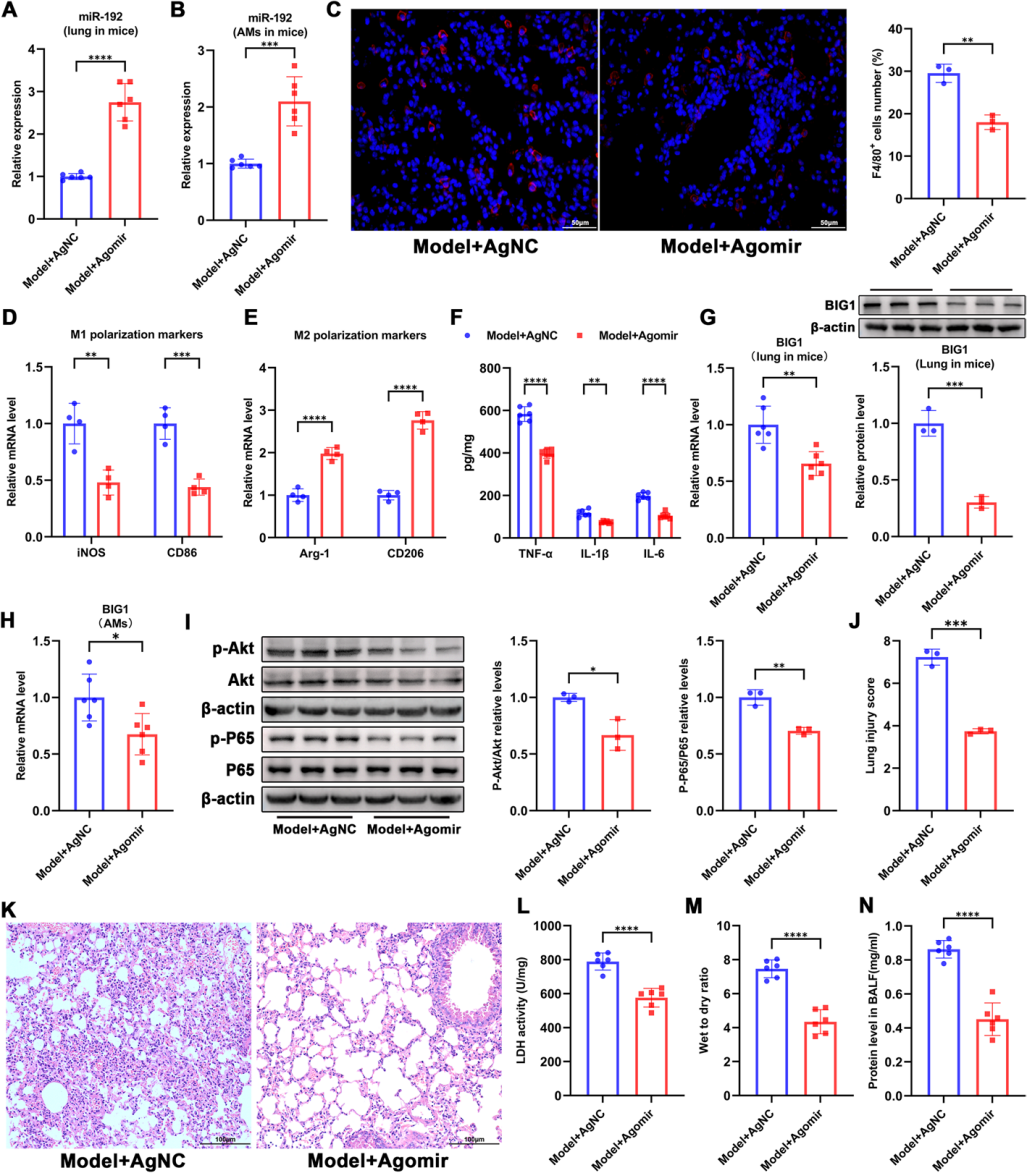
Overexpression of miR-192 alleviates LPS-induced alveolar macrophage activation and inflammation. ALI modeling was performed after intratracheal instillation of miR-192 agomir in obese mice. **A**, **B** RT-qPCR detection of miR-192 expression in mouse lung tissue and alveolar macrophages. **C** Immunofluorescent labeling of macrophages. Macrophages are labeled in red (F4/80) and nuclei in blue (DAPI). F4/80-positive cells were counted using FLOWJO software. Scale bar, 50 µm. **D**, **E** RT-qPCR assessment of mRNA levels of M1 macrophage markers (iNOS and CD86) and M2 macrophage markers (Arg-1 and CD206) in lung tissues. **F** ELISA measurements of protein levels of TNF-α, IL-1β, and IL-6 in lung tissues. **G** RT-qPCR and western blot assessment of BIG1 mRNA and protein expression in mouse lung tissue. **H** RT-qPCR detection of BIG1 mRNA expression in alveolar macrophages. **I** WB assessment of p- AKT, AKT, p-p65, and p65 levels. **J**, **K** H&E staining illustrating the extent of lung pathological damage and corresponding lung injury scores. **L** LDH activity in lung tissue was assessed using an LDH activity assay kit. **M** Wet-to-dry weight ratio of the lungs. **N** Protein concentration in the BALF supernatant was determined using the BCA protein quantitation kit. Data are expressed as the mean ± SD, **P < 0.01. ***P < 0.001. ****P < 0.0001

To validate the role of miR-192 in mice, we instilled miR-192 Agomir into the trachea of obese mice and then induced ALI. Based on the DIO-LPS model (Model group), the Agomir significantly increased the expression of miR-192 in lung tissue and alveolar macrophages (Fig. 8A, B). Immunofluorescence results showed that after overexpression of miR-192, the number of pulmonary macrophages (F4/80^+^) in mice significantly decreased (Fig. 8C). RT-qPCR results indicated that after Agomir treatment in Model mice, the mRNA levels of M1 macrophage markers iNOS and CD86 in lung tissue were significantly reduced, while the levels of M2 macrophage markers Arg-1 and CD206 were significantly increased (Fig. 8D, E). ELISA results revealed that the protein levels of pro-inflammatory factors (TNF-α, IL-1β, and IL-6) in the lung tissue were also significantly reduced (Fig. 8F). In addition, treatment with miR-192 Agomir resulted in decreased expression of BIG1 mRNA and protein in lung tissue (Fig. 8G). Consistent with the trend observed in lung tissue, a significant decrease in BIG1 mRNA expression was also observed in mouse alveolar macrophages (Fig. 8H). Concurrently, Western Blot results indicated that the phosphorylation levels of AKT and NF-κB p65 in lung tissue were significantly reduced after treatment with miR-192 Agomir (Fig. 8I).

Subsequently, we explored the effect of overexpressing miR-192 on the degree of lung injury induced by LPS in obese mice. HE staining showed that after Agomir administration, both the pathological manifestations in the lung and the lung injury scores were significantly improved (Fig. 8J, K). Meanwhile, LDH activity in lung tissue, W/D ratio, and protein concentration in BALF from the Agomir group were also significantly reduced (Fig. 8L–N). These findings suggest that overexpression of miR-192 can alleviate LPS-induced pulmonary macrophage activation and ALI in obese mice.

## Discussion

In the foreseeable future, obesity will remain a prominent global health concern. It serves as a primary risk factor for ALI/ARDS, amplifying the effects of various ALI models, consistent with our research findings [5–8]. Despite the extensive research on ALI/ARDS, studies specifically addressing ARDS in obese individuals are scarce. Investigating the molecular mechanisms through which obesity aggravates ALI/ARDS holds the potential to advance ARDS treatments tailored to obese patients. Macrophages, recognized as the predominant immune cells within the pulmonary milieu, play a fundamental role in the development of acute lung injury, as extensively documented [22, 27, 36]. However, the specific involvement of macrophages in the context of obesity-related ALI/ARDS has yet to be addressed. Our study observed a significant increase in macrophage numbers and pro-inflammatory polarization in the lung tissues of obese mice. To gain deeper insights into the precise contribution of macrophages to the exacerbation of LPS-induced ALI in obese mice, we conducted an experiment involving the depletion of pulmonary macrophages via intratracheal administration of clodronate liposomes. Our findings demonstrate that the removal of macrophages in obese mice ameliorates lung inflammation and injury, irrespective of exposure to LPS. Our research is the first to underscore the pivotal role of macrophages in exacerbating LPS-induced ALI during obesity, presenting a compelling case for the therapeutic potential of targeting macrophages in mitigating obesity-related ALI.

MiRNA dysregulation is closely linked to a variety of human diseases [37]. Our study revealed a significant downregulation of miR-192 expression in the lung tissues of obese patients, which exhibited a negative correlation with M1 macrophage activation markers, including iNOS and TNF-α. This suggests a potential association between miR-192 and macrophage activation during obesity. Similarly, consistent expression patterns were observed in lung tissues and alveolar macrophages of high-fat diet (HFD)-induced obese mice, regardless of LPS exposure. Upon treating MH-S cells with palmitic acid (PA), used to simulate the metabolic stress of obesity, miR-192 expression significantly decreased. Furthermore, this reduction was accentuated with LPS stimulation. Several studies have indicated the involvement of miR-192 in macrophage activation and inflammation. For instance, a microarray analysis by Hirotake and colleagues revealed that miR-192 substantially suppresses the expression of pro-inflammatory cytokines, including IL-6 and CCL2, in LPS-stimulated macrophages [18]. Another study demonstrated that miR-192 promotes the expression of the M2 macrophage phenotype in vitro while inhibiting the polarization of the M1 phenotype in mouse bone marrow-derived macrophages (BMDM) [38]. Considering the dysregulation of miR-192 in obesity and its profound impact on macrophage activation, we further explored its role in LPS-induced ALI in obese mice, both in vitro and in vivo. Our results showed that inhibiting miR-192 in MH-S cells amplified LPS-induced macrophage pro-inflammatory polarization, cytokine secretion, and activation of pro-inflammatory signaling pathways. Conversely, the overexpression of miR-192 significantly inhibited macrophage activation and inflammation induced by PA and LPS. In our in vivo experiments, the intratracheal instillation of miR-192 agomir led to a marked upregulation of miR-192 expression in lung tissues and alveolar macrophages. Overexpressing miR-192 ameliorated macrophage activation, lung injury severity, and inflammation in LPS-induced obese mouse lung tissues. Notably, alveolar macrophages, being the "first line of defense" in the respiratory system, possess rapid and efficient phagocytic capabilities against inhaled particles and pathogens [39]. These findings suggest that targeting alveolar macrophages through intratracheal drug delivery represents a promising and potent therapeutic approach. In summary, our results demonstrate obesity-induced downregulation of miR-192 expression exacerbates LPS-induced ALI by promoting macrophage activation.

We delved further into the molecular mechanism by which miR-192 regulates macrophage activation, focusing on its downstream targets. Through a comprehensive analysis employing bioinformatics tools, dual-luciferase reporter assays, RT-qPCR, and Western blot assays, we successfully identified ARFGEF1, also known as Brefeldin A-inhibited guanine nucleotide-exchange protein 1 (BIG1), as a direct target of miR-192. BIG1 (ARFGEF1) is a high molecular weight ADP-ribosylation factor-specific guanine nucleotide-exchange factor with a crucial role in membrane transport [34]. Several studies have underscored its significance in macrophage inflammation [34, 35, 40, 41]. For example, You and colleagues demonstrated that BIG1 promotes the production of inflammatory cytokines in microglia cells in response to LPS, mediated by the PI3K/AKT/NF-κB signaling pathway [41]. Similarly, Liu and others revealed that the absence of BIG1 in macrophages can suppress the inflammatory response induced by LPS, and myeloid-specific BIG1 knockout (BIG1 cKO) confers protection against septic injuries in mice [34]. Intriguingly, BIG1 can also modulate sepsis-induced lung injury by affecting lipid raft-dependent macrophage inflammatory responses [35]. Our findings suggest that the overexpression of BIG1 can counteract the protective effects of miR-192 on macrophage activation and inflammation, thereby highlighting miR-192’s mechanism of action in targeting BIG1 within MH-S cells.

N6-methyladenosine (m6A) modification is the most common internal modification found in eukaryotic mRNA and non-coding RNA [42]. It is a dynamic and reversible process, primarily regulated by ‘writers’ that add methyl groups (such as METTL3, METTL14, and WTAP), ‘erasers’ that remove methyl groups (such as FTO and ALKBH5), and ‘readers’ that recognize m6A modifications (such as YTHDF1 and YTHDC2) [42]. m6A modification plays a pivotal role in miRNA maturation [30]. A decline in m6A levels can impede the processing of pri-miRNAs into pre-miRNAs, eventually resulting in reduced mature miRNAs [30–32]. In our research, we detected a decrease in pre-miR-192 alongside an increase in pri-miR-192 in the lung tissues and alveolar macrophages of obese mice, suggesting a possible connection with m6A modification. Subsequent m6A level tests and RIP experiments substantiated our hypothesis. The fat mass and obesity-associated (FTO) protein, the first-known enzyme with the capability to remove m6A modifications [43], is also encoded by the first gene found to be significantly associated with obesity risk through genome-wide association studies [44]. We observed a noteworthy increase in the protein levels of FTO in the lung tissues and alveolar macrophages of obese mice. Additionally, upon treatment with PA, the protein levels of FTO in MH-S cells was reduced and further diminished after LPS stimulation. Consistent with our findings, several studies have found that FTO exhibits dysregulated expression in various tissues of obese individuals and plays a role in mediating obesity-related diseases [45–47]. A methylated RNA immunoprecipitation sequencing (MeRIP-seq) study unveiled that FTO knockdown affects the steady-state levels of several miRNAs [48]. Previous research has also demonstrated that FTO can exert effects in various cancers and inflammatory diseases by influencing miRNA maturation [49, 50]. In our findings, overexpression of FTO in MH-S cells resulted in the downregulation of mature miR-192 and pre-miR-192 expression, concomitant with an accumulation of pri-miR-192. Furthermore, RIP experiments further confirmed that FTO had a similar effect to obesity, and significantly reduced m6A-modified pri-miR-192 after overexpression. Overall, our study elucidates the association between the downregulation of miR-192 expression and m6A demethylation induced by obesity. The role of m6A modification in obesity-related ALI/ARDS warrants further investigation.

## Conclusions

In summary, our study suggests that macrophages are key mediators of obesity-aggravated LPS-induced ALI, and reveals that obesity-induced down-regulation of miR-192 aggravates LPS-induced ALI by promoting macrophage activation. In addition, reduced miR-192 production in obesity is associated with FTO-mediated m6A demethylation of pri-miR-192. miR-192 inhibits pro-inflammatory activation of macrophages by inhibiting AKT/NF-κB pathway by targeting BIG1. These results suggest that targeting macrophages and miR-192 may provide a new therapeutic avenue for obesity-associated ALI.

### Supplementary Information

**Additional file 1.** **Sup Fig. 1.** Decreased activity of miR-192 in HFD lungs. A Cumulative distribution curves represent miR-192 activity. A rightward shift of miR-192 targets (red) indicates a decrease in miR-192 activity (HFD vs. Control), n = 3. Statistical analysis was executed using a two-sided Kolmogorov-Smirnov (KS) test. **Sup. Fig 2.** ARFGEF1 (BIG1) is a potential target of miR-192. A Potential targets of miR-192 were predicted by integrating results from three databases: TargetScan, miRDB, and miRWalk. B Conservation of the miR-192 target sequence in the ARFGEF1 3' UTR across different species, as well as the conservation of the miR-192 sequence itself among various species. **Table S1.** Patient Demographics & Clinical Characteristics. **Table S2.** Agomir and antagomir sequences used in this study. **Table S3.** Primers sequences for qRT-PCR used in this study.

## Data Availability

The dataset supporting the conclusions of this article is available from the corresponding author upon reasonable request. The microarray data has been deposited in the NCBI’s Gene Expression Omnibus repository and are accessible via GEO series accession number GSE229262. However, the dataset is currently private and scheduled to be publicly available on Apr 09, 2024.

## Abbreviations

3ʹ-UTR: 3ʹ-Untranslated region
ALI: Acute lung injury
ALKBH5: AlkB homolog 5
AMs: Alveolar macrophages
Arg-1: Arginase 1
ARDS: Acute respiratory distress syndrome
BIG1: Brefeldin A-inhibited guanine nucleotide-exchange protein 1
BMI: Body mass index
DIO: Diet-induced obesity
FTO: Fat mass and obesity-associated
HFD: High-fat diet
iNOS: Inducible nitric-oxide synthase
LDH: Lactate dehydrogenase
LPS: Lipopolysaccharide
m6A: N6-methyladenosine
METTL3: Methyltransferase Like 3
METTL14: Methyltransferase Like 14
MH-S: Mouse alveolar macrophages
PA: Palmitic acid
WHO: World Health Organization
WTAP: Wilms tumor 1 associating protein
YTHDC2: YTH domain containing
YTHDF1: YTH N6-methyladenosine RNA binding protein
