# Supplementary material for: Obesity-induced downregulation of miR-192 exacerbates lipopolysaccharide-induced acute lung injury by promoting macrophage activation
Source: Cell Mol Biol Lett. 2024 Mar 14;29:36. doi: 10.1186/s11658-024-00558-w (PMC10938800; doi:10.1186/s11658-024-00558-w)
Supplement: Supplementary file 1 — Additional file 1. Sup Fig. 1. Decreased activity of miR-192 in HFD lungs. A Cumulative distribution curves represent miR-192 activity. A rightward shift of miR-192 targets (red) indicates a decrease in miR-192 activity (HFD vs. Control), n = 3. Statistical analysis was executed using a two-sided Kolmogorov-Smirnov (KS) test. Sup. Fig 2. ARFGEF1 (BIG1) is a potential target of miR-192. A Potential targets of miR-192 were predicted by integrating results from three databases: TargetScan, miRDB, and miRWalk. B Conservation of the miR-192 target sequence in the ARFGEF1 3' UTR across different species, as well as the conservation of the miR-192 sequence itself among various species. Table S1. Patient Demographics & Clinical Characteristics. Table S2. Agomir and antagomir sequences used in this study. Table S3. Primers sequences for qRT-PCR used in this study. [file 11658_2024_558_MOESM1_ESM.docx]

**Obesity-induced downregulation of miR-192 exacerbates lipopolysaccharide-induced acute lung injury by promoting macrophage activation.**

**Figures**


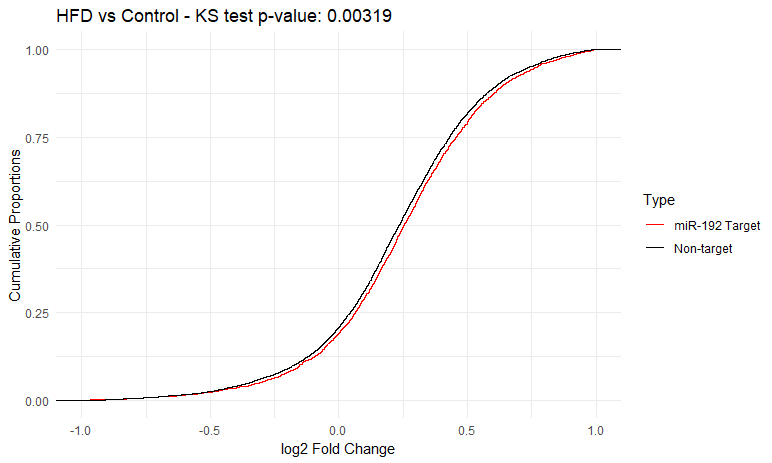


**Fig S1. Decreased activity of miR-192 in HFD lungs.** (A)Cumulative distribution curves represent miR-192 activity. A rightward shift of miR-192 targets (red) indicates a decrease in miR-192 activity (HFD vs. Control), n=3. Statistical analysis was executed using a two-sided Kolmogorov-Smirnov (KS) test.


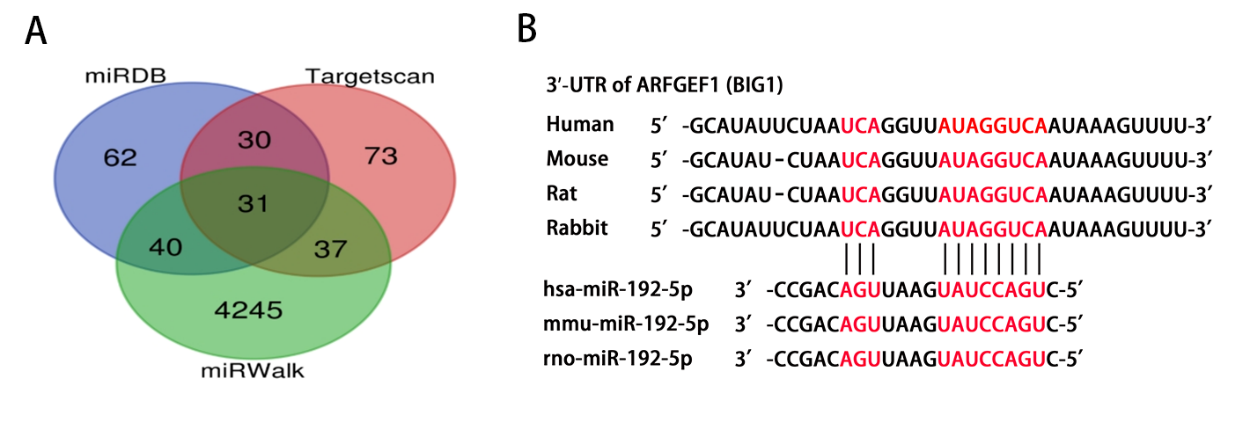


**Fig S2. ARFGEF1 (BIG1) is a potential target of miR-192.** (A) Potential targets of miR-192 were predicted by integrating results from three databases: TargetScan, miRDB, and miRWalk. (B) Conservation of the miR-192 target sequence in the ARFGEF1 3' UTR across different species, as well as the conservation of the miR-192 sequence itself among various species.

**Table S1.** **Patient Demographics & Clinical Characteristics**

| Parameter | Control Group  (BMI 18.5-24.9 kg/m²) | Obesity Group  (BMI≥30.0 kg/m²) |
| --- | --- | --- |
| Number of Patients | 4 | 8 |
| Age (years) | 52.5 ± 6.1644 | 54.375 ± 7.7172 |
| Gender | 2 Males / 2 Females | 3 Males / 5 Females |
| BMI (kg/m²) | 23.3 ± 0.2487 | 32.88875 ± 1.1642 |
| Surgery Reason | 1 Solitary pulmonary nodules | 3 Solitary pulmonary nodules |
|  | 3 Lung neoplasms | 5 Lung neoplasms |
| Distance to Nodule/Tumor (cm) | >5cm | >5cm |

BMI, body mass index. Data are expressed as the mean ± SD.

**Table S2. Agomir and antagomir sequences used in this study.**

| Gene(mouse) | Sequences (5’-3’) |
| --- | --- |
| miR-192-5p agomir | CUGACCUAUGAAUUGACAGCC |
|  | CUGUCAAUUCAUAGGUCAGUU |
| Agomir-NC | UUCUCCGAACGUGUCACGUTT |
|  | ACGUGACACGUUCGGAGAATT |
| miR-192-5p antagomir | GGCUGUCAAUUCAUAGGUCAG |
| Antagomir-NC | CAGUACUUUUGUGUAGUACAA |

**Table S3. Primers sequences for qRT-PCR used in this study.**

| Gene | Sequences (5’-3’) | |
| --- | --- | --- |
| **Mouse** |  |  |
| β-actin | F: AGATTACTGCTCTGGCTCCTAGC | R: ACTCATCGTACTCCTGCTTGCT |
| iNOS | F: TGGAGCGAGTTGTGGATTGT | R: TCTCTGCCTATCCGTCTCGTC |
| CD86 | F: ATCCAAGAGCCACTCCTACCT | R: TCCAGACCTTTCCAGGCATTT |
| Arg-1 | F: GCATATCTGCCAAAGACATCGT | R: CCATCACCTTGCCAATCCC |
| CD206 | F: AGAGCTGGCGAGCATCAAGAG | R: TTCCATAGGTCAGTCCCAACCAA |
| BIG1 | F: ACGTGTAACTGCACCCTGG | R: TGATGGAGATGGGGGTTCG |
| FTO | F: CTGAGGATGAAAGTGAGGACGAGT | R: TGGTGGGTGGCATTGAGGTC |
| METTL3 | F: ATCCAGGCCCATAAGAAACAG | R: CTATCACTACGGAAGGTTGGG |
| METTL14 | F: CTGAGAGTGCGGATAGCATTG | R: GAGCAGATGTATCATAGGAAGCC |
| WTAP | F: AACAATGGTAGACCCAGCAATC | R:AGGCGTAAACTTCCAGGCAC |
| ALKBH5 | F: CAGTGGGTATGCTGCTGATG | R: GGGTCTCTGGTGTTTCCTGA |
| YTHDF1 | F: CTGCAGTTAAGACGGTGGGT | R: TAGCAATGGCTGCCCATGAA |
| YTHDC2 | F: GGTCCGATCAATCATCTGT | R: GAAGTAACGAATAGGCATGT |
| pri-miR-192 | F: GCATGGGAAGGCAACGAAT | R: GCAGCCAGAGGAGAGAAAAGAGT |
| pre-miR-192 | F: GGGCTCTGACCTATGAATTGACA | R: ACCTGTGACCTATGGAATTGGC |
| U6 | F: GGAACGATACAGAGAAGATTAGC | R: TGGAACGCTTCACGAATTTGCG |
| miR-192-5p | F: CTGACCTATGAATTGACAGCC | |
| **Human** |  |  |
| β-actin | F: TGGCACCCAGCACAATGAA | R: CTAAGTCATAGTCCGCCTAGAAGCA |
| iNOS | F: ACTGGATTTGGCTGGTCCCT | R: CTCTTGGGTCTCCGCTTCTC |
| TNF-α | F: TGGGCAGGTCTACTTTGGGA | R: GAGGTTGAGGGTGTCTGAAGG |
| miR-192-5p | F: TGACCTATGAATTGACAGCC | |
